# A Policy-Driven Approach to Secure Extraction of COVID-19 Data From Research Papers

**DOI:** 10.3389/fdata.2021.701966

**Published:** 2021-08-12

**Authors:** Lavanya Elluri, Aritran Piplai, Anantaa Kotal, Anupam Joshi, Karuna Pande Joshi

**Affiliations:** ^1^ IS Department, University of Maryland Baltimore County, Baltimore, MD, United States; ^2^ CSEE Department, University of Maryland Baltimore County, Baltimore, MD, United States

**Keywords:** COVID-19, knowledge graph, privacy, UMLS, HIPAA

## Abstract

The entire scientific and academic community has been mobilized to gain a better understanding of the COVID-19 disease and its impact on humanity. Most research related to COVID-19 needs to analyze large amounts of data in very little time. This urgency has made Big Data Analysis, and related questions around the privacy and security of the data, an extremely important part of research in the COVID-19 era. The White House OSTP has, for example, released a large dataset of papers related to COVID research from which the research community can extract knowledge and information. We show an example system with a machine learning-based knowledge extractor which draws out key medical information from COVID-19 related academic research papers. We represent this knowledge in a Knowledge Graph that uses the Unified Medical Language System (UMLS). However, publicly available studies rely on dataset that might have sensitive data. Extracting information from academic papers can potentially leak sensitive data, and protecting the security and privacy of this data is equally important. In this paper, we address the key challenges around the privacy and security of such information extraction and analysis systems. Policy regulations like HIPAA have updated the guidelines to access data, specifically, data related to COVID-19, securely. In the US, healthcare providers must also comply with the Office of Civil Rights (OCR) rules to protect data integrity in matters like plasma donation, media access to health care data, telehealth communications, etc. Privacy policies are typically short and unstructured HTML or PDF documents. We have created a framework to extract relevant knowledge from the health centers’ policy documents and also represent these as a knowledge graph. Our framework helps to understand the extent to which individual provider policies comply with regulations and define access control policies that enforce the regulation rules on data in the knowledge graph extracted from COVID-related papers. Along with being compliant, privacy policies must also be transparent and easily understood by the clients. We analyze the relative readability of healthcare privacy policies and discuss the impact. In this paper, we develop a framework for access control decisions that uses policy compliance information to securely retrieve COVID data. We show how policy compliance information can be used to restrict access to COVID-19 data and information extracted from research papers.

## Introduction

The COVID-19 pandemic is one of the most important global events in recent history. It has impacted human society at every level and created challenges to public health administration, medical research, and patient data management. It has led to significant cooperation among the scientific community to understand the disease and look for cures quickly. Medical researchers have released an unprecedented amount of data related to COVID-19 to understand the disease better. For example, the White House OSTP has released a large dataset of papers being published related to COVID research from which the research community is extracting knowledge and information. It is encouraging to see the global research community motivated to address the concerns of COVID-19. Data collection, data storage, data analytics, and data sharing are key to overcoming the pandemic. However, this wide sharing of potentially sensitive data raises critical questions about privacy and security. We address these concerns in this paper. We show a simple system that can extract useful information from papers, and express it in a knowledge graph (represented in OWL). We then show how policies controlling access can similarly be extracted from text descriptions and encoded in a knowledge graph to limit what can be done with the extracted knowledge.

Published articles on COVID-19 have a crucial role in our understanding of the pandemic. There are 23,000 + unique published articles indexed on Web of Science and Scopus between 1 January and 30 June 2020 (da Silva et al., 2020). Gathering relevant information from the large collection of published articles is a difficult task. It is time-intensive to read and investigate key published articles manually. As the global community is rushing to find a pandemic solution, we need a more efficient way to extract key information from COVID-19 related research papers. Approaches from Text Analysis and NLP are being developed to automatically read papers and extract key knowledge. In this paper, we show a prototype system with a machine learning-based knowledge extractor, which draws out key medical information from COVID-19 related academic research papers. We represent this knowledge in a Biomedical Knowledge Graph (BKG) that extends the information captured in the standard Unified Medical Language System (UMLS).

We used an established pipeline (Piplai et al., 2020a; Piplai et al., 2020b) for knowledge extraction, but retrained it for the medical domain, and populated the BKG that contains information from research papers on COVID-19. We used the UMLS (Bodenreider, 2004) to develop the knowledge graph schema. We also added necessary classes to define the sources for the data used in the medical experiments. This would help users search for the data sources that lead to the information present in the research paper and the BKG. They can also learn about the data collection methods’ privacy compliance from a related knowledge graph. This is described in greater detail in *Extracting COVID-19 Knowledge From Published Research Paper*.

While it is vital to share data related to COVID-19, including data about patients, treatments, and outcomes, it is necessary to ensure that this data is secured. Any analysis respect associated security and privacy policies. Ensuring security and privacy while processing done on shared data should the data and handling patient records has become a primary challenge, which we address in this paper. We propose a system to restrict access to controlled data. We use published paper and HIPAA regulation as an example to demonstrate the proposed framework. The Health Insurance Portability and Accountability Act (HIPAA) (for Disease Control, C., Prevention, 2003) regulates the security and privacy of the data retained by the healthcare providers in the US. It has provided specific guidance for COVID-19 data. All COVID-19 patient records in the United States must comply with the new rules in HIPAA. The HIPAA COVID-19 privacy Rule (OCR, 2020a) provides guidelines to securely access personally identifiable information (PII) of patients who have been affected or exposed to COVID-19. The regulation also specifies the guidance for contacting former COVID-19 patients for plasma donation (OCR, 2020c). It further establishes the rules for disclosing personal health information to media (OCR, 2020b). The law also addresses remote telehealth communication-related questions for COVID-19 patients (Lee et al., 2020). We would like to automatically ensure that any analysis done abides by these and other rules about sensitive medical data.

We developed a knowledge graph (and an associated ontology) to define COVID-related privacy and security rules, such as those detailed in HIPAA. This ontology extends our previous work in creating a HIPAA ontology for automatically populating HIPAA rules to access patient records (Joshi et al., 2016). It helps distinguish healthcare domain-specific privacy and security measures. Our previous HIPAA ontology identifies concepts specified in the regulation not related to COVID. By expanding this ontology combined with COVID rules and integrating the HIPAA and COVID compliance guidelines, data sources (e.g., healthcare providers) and data analysts can quickly check and enforce HIPAA and COVID privacy requirements. We describe the enhanced and updated ontology in *Developing HIPAA Ontology*. Health centers or organizations utilizing COVID-19 patient data can use this ontology to ensure their privacy policies have all the rules stated by HIPAA-COVID compliance. The semantically rich, machine-processable knowledge graph developed using our methodology captures all the rules stated in HIPAA and COVID guidance. It can also help identify missing rules in the organization’s privacy policy, which can then be added as needed.

Privacy Policies also need to be understandable to the average user. Clients/patients should not have to agree to rules and obligations that they do not fully understand. The privacy policy should be unambiguous and easy to read. In the previous work by our group Kotal et al. (2020), we studied trends in privacy policies of popular e-services and developed a metric to measure the vagueness in such policies. We used the same model to measure the textual quality of privacy policies for organizations that collect, store, and/or use patient data related to COVID. Along with the regulation compliance study, this can help organizations create privacy policies that are comprehensive and useful for the reader.

In *Introduction* we explained the motive for this work and in *Related Work* we talk about the background and related work in this area. In *Framework to Securely Access COVID-19 Data*, we describe our methodology of building the HIPAA COVID knowledge graph and detail the ontology we have developed using OWL. Also, In this section, we explain the NLP approaches took to obtain the rules and populate policy documents of various healthcare providers as instances of our ontology and present the results obtained from our validation. We end with the conclusions and future work in *Conclusion and Future Work*.

## Related Work

In this paper, we show proof of work of a pipeline that can extract key information from published papers and in doing that point out any privacy vulnerabilities in the data sharing process. In our pipeline, we parsed published articles on COVID-19 and privacy policy documents of healthcare centers. We extracted knowledge from documents in natural language and represented them in a machine-processable, semantic framework. The key techniques that help us in extracting and representing knowledge in published documents and policy articles are Named Entity Recognition (NER) and Knowledge Graph (KG). In this section, we discuss prior work related to these methods.

### Named Entity Recognition

In this section, we discuss how NER has been used previously for Information Extraction. Identifying information units like names, organization, location, time, date, etc. is critical to the task of information extraction. Named entity recognition can be broadly defined as the task of identifying references to these entities in the text (Nadeau and Sekine, 2007). NER has been used for the task of entity extraction in various domains including cybersecurity (Dasgupta et al., 2020), law (Dozier et al., 2010), biology (He and Kayaalp, 2008) etc. In our previous work in the cybersecurity domain (Piplai et al., 2020b), we described a pipeline to represent and model CTI. We then used Stanford NER (Manning et al., 2014) and.

Regular Expressions to detect cyber-entities from the open-source text. Mittal et al. in their paper (Mittal et al., 2016), also used NER to automatically generate alerts from Twitter feeds relevant to cybersecurity. Stanford CoreNLP toolkit (Manning et al., 2014) is an extensible pipeline that provides core NER analysis. This toolkit is widely used in research and commercial organizations for information and extraction. To use the Stanford CoreNLP toolkit in the medical domain we needed a structured medical vocabulary and a dataset of medical texts annotated within the vocabulary. The Unified Medical Language System (UMLS) (Bodenreider, 2004) is a repository of biomedical vocabularies developed by the US National Library of Medicine. The UMLS integrates over two million names for some 900,000 concepts from more than 60 families of biomedical vocabularies, as well as 12 million relations among these concepts. Mohan and Li (2019) developed a training dataset for biomedical entity extraction that uses UMLS as the target ontology.

### Knowledge Graph

A knowledge graph is a set of semantic triples, which are pairs of “entities” with “relationships” between them. It is useful for feeding intelligent systems and agents with formalized knowledge of the world (Paulheim, 2017). Knowledge graphs can be refined to contain knowledge about a specific domain. In our prior work, we used Cybersecurity Knowledge Graphs (CKGs) to represent Cyber Threat Intelligence (CTI) (Piplai et al., 2020a; Piplai et al., 2020b; Piplai et al., 2020c). To build a Knowledge Graph specific to a domain, we need to define the ontology schema and entity relations in the domain. In our prior work, we created an ontology schema to extract and represent knowledge in GDPR and PCI DSS (Elluri et al., 2018), and cloud privacy policies (Joshi et al., 2020). Joshi et al. (2016), Kim and Joshi (2021) also defined an ontology to extract knowledge from HIPAA regulation, before the COVID-19 updates. In our pipeline, we extended the pre-COVID HIPAA ontology to include regulations that were added to address COVID-19. We used this ontology schema to create a Knowledge Graph for regulation compliance of healthcare privacy policy. To extract knowledge from medical articles on COVID-19, we used the entity-relations described in UMLS.

## Framework to Securely Access COVID-19 Data

We developed a framework that can extract key information from published papers, identify privacy vulnerabilities in the data sharing process and allow access to securely extracted COVID-19 data. There are three key steps to securely accessing COVID-19 information. They are as follows:

1. Extracting COVID-19 Knowledge from Published Research Paper.
2. Extracting Privacy Compliance Requirements from HIPAA COVID-19 and Organizational Privacy Policies.
3. Making Access Control decisions to securely retrieve COVID-19 data.

**FIGURE 1:**
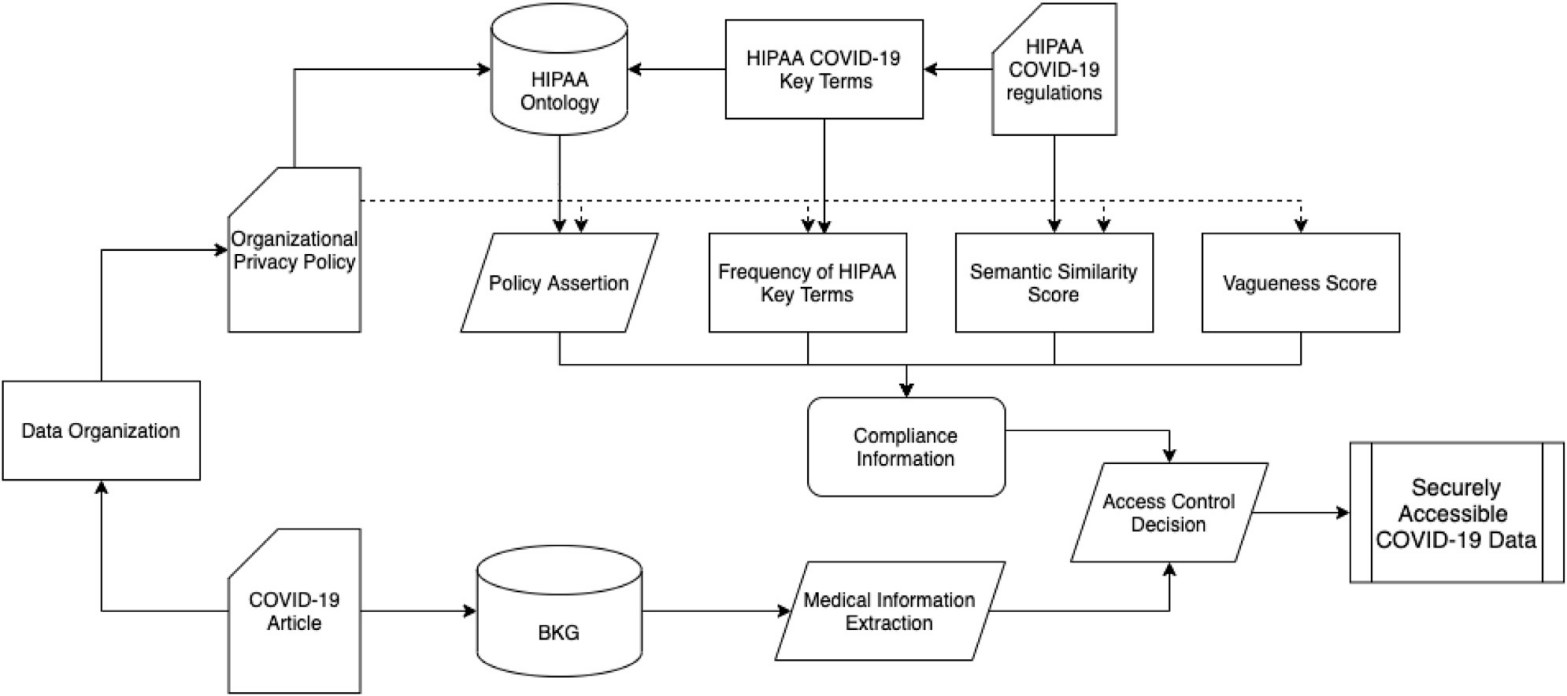
Architecture flow to securely access COVID-19 data from published research paper.

Figure 1 shows the overall architecture of our framework. In the following section, we describe the details of our framework and demonstrate how it works. *Extracting COVID-19 Knowledge From Published Research Paper* describes our method to create a Biomedical Knowledge Graph (BKG) with COVID-19 data. In *Extracting Privacy Compliance Requirements From HIPAA COVID-19 and Organizational Privacy Policies*, we give a brief description of the stages to extract compliance information for organizational privacy policies. We describe each stage in details in *Developing HIPAA Ontology* and *Generating Compliance Information*. In *Making Access Control Decisions to Securely Retrieve COVID-19 Data*, we describe how we can use compliance information to make access control decisions and allow secure access to COVID-19 data.

### Extracting COVID-19 Knowledge From Published Research Paper

We extracted knowledge from a published research paper on COVID-19 into a Biomedical Knowledge Graph (BKG). In this section, we discuss the different components of the knowledge extraction pipeline that lead to the generation of the BKG. We also show how this BKG can be queried to extract COVID-19 information. Representing unstructured data in the form of a knowledge graph helps to extract important information from the data and derive relationships between them. This also helps end-users to query the knowledge graph and retrieve information without having to go through the data manually. We mined information from unstructured research papers, written in natural language, about COVID-19. We presented the mined information in a knowledge graph (BKG) that has reasoning capabilities and also an interface to query the populated BKG.

#### BKG Schema

**FIGURE 2:**
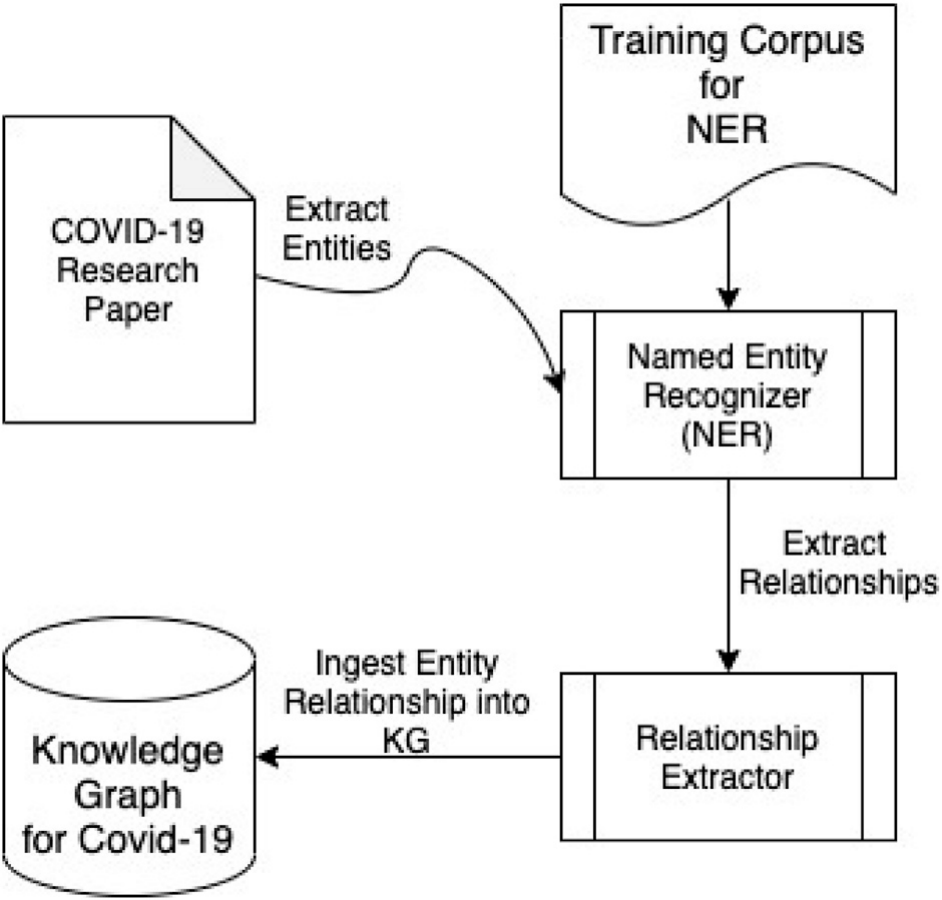
Architecture flow to extract knowledge from medical research articles.

The schema of our BKG is based on the UMLS. This is a well-recognized ontology for the medical domain, as it defines classes for medical entities and the possible relationships that can exist between them that are accepted by the medical community. We extended the UMLS schema, by adding another class called “Data Source” that helps in representing the origin of various facts present in the research paper. We also added necessary relationships to support the addition of the aforementioned class. The information extraction pipeline is based on a knowledge extraction pipeline that members of our team developed for cybersecurity. It consists of a Named Entity Recognizer (NER) that classifies words or groups of words to a particular entity class present in our modified UMLS-based BKG. This results in a set of entities and their corresponding entity-class labels. The next stage is the relationship extractor, which takes pairs of entities that have credible relationships between them and produces a relationship label as an output. We then take our entity-relationship set and assert that into our BKG. Figure 2 describes the different components of our pipeline.

#### NER and RelExt for BKG

In our prior works (Dasgupta et al., 2020; Piplai et al., 2020b), we have described different NER strategies in the domain of cybersecurity. We reuse the NER model that was described by Piplai et al. (2020b). This NER model is based on Stanford NER that used Conditional Random Fields and Gibbs’ sampling for the NER task. We used a public dataset (Mohan and Li, 2019) that consisted of 107819 words and was annotated with UMLS classes. We also annotated the training set with an additional class called the “data source.” A total of 124 UMLS classes (including the additional class “Data source”) were used in our BKG. We trained the model for 343 iterations and used the trained model on COVID-19 research papers to identify the key medical terms and expressions. At the end of this stage, we are left with a list of extracted entities and their corresponding entity types that includes the newly added class.

The next stage of the pipeline is the Relationship Extractor. The relationship extractor takes pairs of entities and establishes a relationship between them. Since UMLS provides the entire schema for the ontology, we also have a list of possible relationships that can exist between pairs of entity classes. We used this to pre-process the candidate entity pairs that we provide as an input to the relationship extractor. We discarded pairs of entities that do not have a credible relationship between them according to our UMLS-based schema. We provide the rest as input to the Relationship Extractor. The Relationship extractor is a four layered neural network that takes the word2vec (Mikolov et al., 2013) embeddings of the two candidate entities. We have a list of 46 relationships specified by our schema. We also have an additional class that signifies “no relationship.” The word2vec embedding has a dimension of 200. Two entities create a 400-dimensional input vector for the neural network. We then have three hidden layers of dimensions 200, 100, and 100 respectively. We have a final softmax layer that has 47 dimensions.

#### Querying BKG to Retrieve COVID-19 Information

**FIGURE 3:**
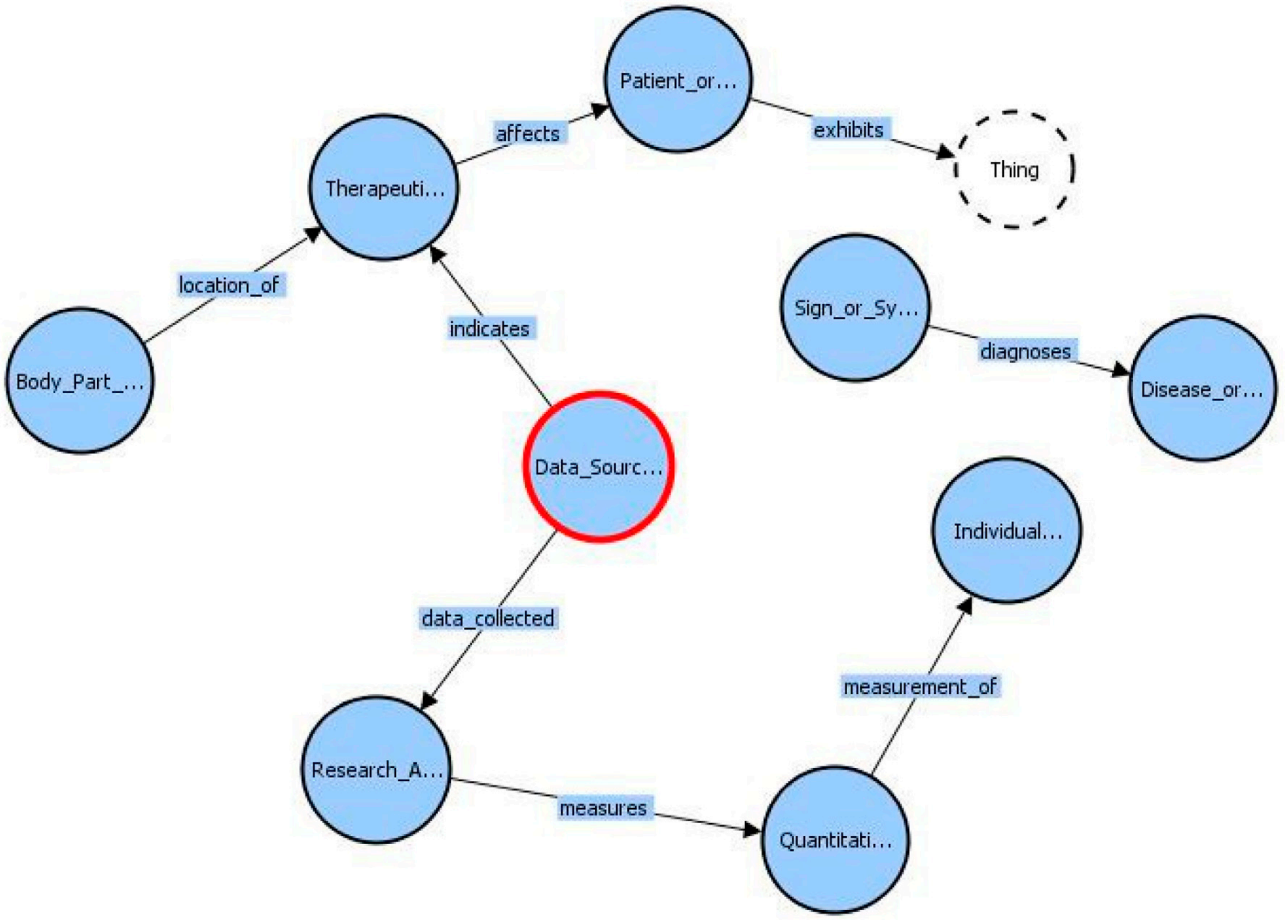
A subset of our BKG schema as represented by the VOWL visualizer.

At this stage, we have an entity-relationship set that not only captures the information present in COVID-19 research papers but also associates the source of knowledge for the facts present in those papers. We use RDFLib, a Python library, to dynamically create a knowledge graph instance and populate them. Figure 3 describes a subset of classes and relationships that can exist between them. The classes are represented by circles and the relationships are represented by directed lines signifying the “domain” and “range” of the relationship. The class “Data Source” that has been added by us to include additional information, is marked by a red circle. We can see that this Class “indicates” a “Therapeutic or Preventive Measure.” “Data Source” also has an additional relationship called “data collected” with a class called “Research Activity.”

**FIGURE 4:**
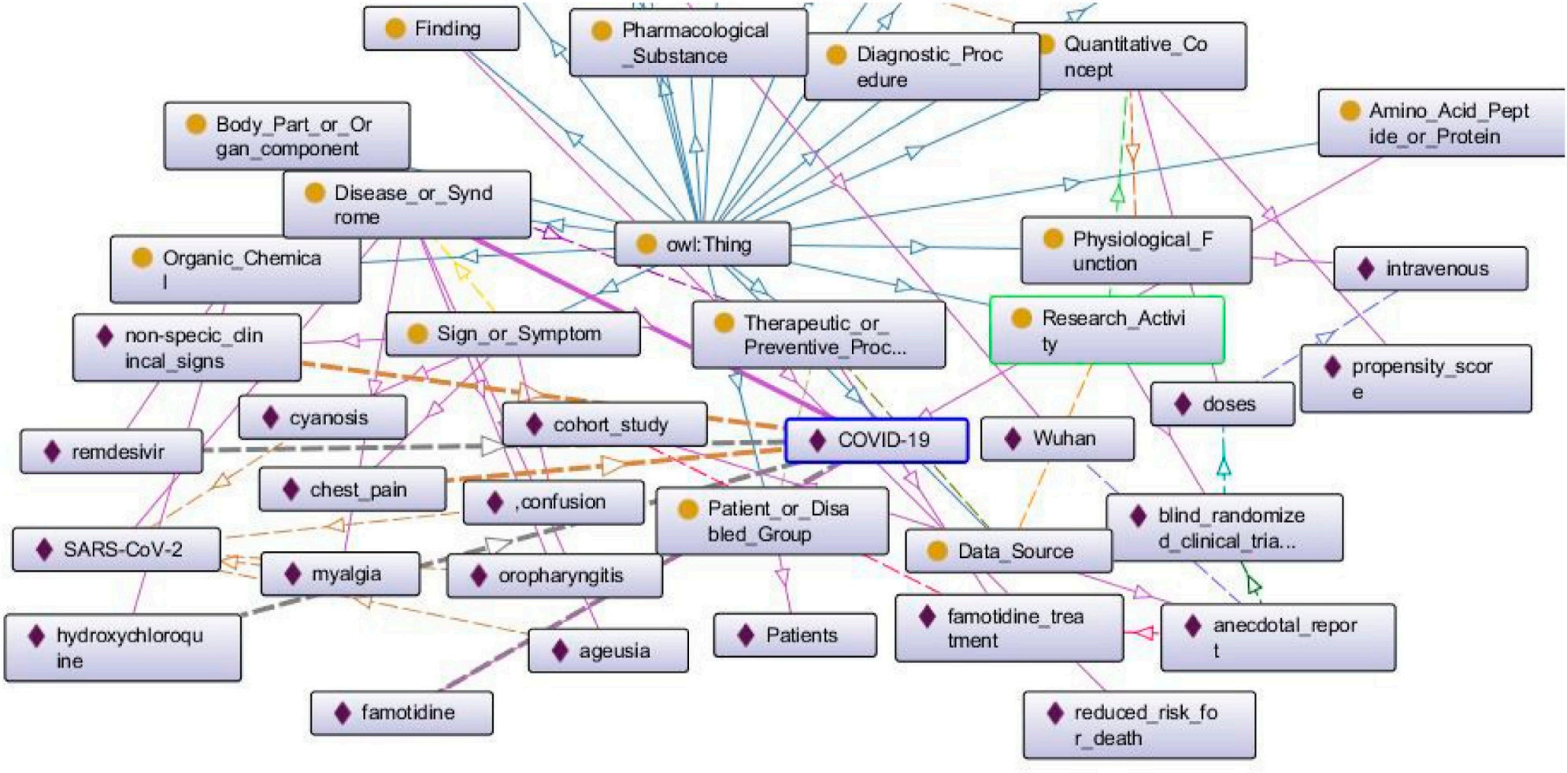
A populated BKG from one research paper about COVID-19. We discuss this graph in *Querying BKG to Retrieve COVID-19 Information*.

In Figure 4, we can see a part of a BKG populated from an early research paper about COVID-19 (Malone et al., 2021). The rectangles with yellow circles on them indicate classes, and the rectangles with purple rhombuses on them indicate the entities. The arrows are color-coded and they represent individual relationships that exist between pairs of entities. The blue arrows going upwards signify the relationship “subclass of” that exists between all classes and the superclass “owl: Thing.” We see a few bold lines that signify all the relationships that exist for the entity “COVID-19.” This entity has been identified as a “Disease or Syndrome” as is manifested by the bold purple line that connects the class with the entity. The dotted yellow arrows that exist between “COVID-19” and “non-specific clinical signs,” “chest pain” respectively indicate the relationship “diagnoses.” The bold grey dotted lines that exist between “COVID-19” and “remdesevir,” “hydroxychloroquine,” “famotidine” respectively signify the relationship “treats.”

Next, we demonstrate some of the reasoning capabilities of the BKG with the help of some SPARQL queries. For example, if we are interested to know what data source indicates a therapeutic or preventive procedure, we run the following query. The query translates to “Find all pairs of entities such that one of them is a Data Source, the other is a Therapeutic or Preventive Procedure, and the Data Source indicates the Procedure.” The variables “x” and “y” indicate the particular entities we are interested in retrieving from the BKG. All the entity types and property or relationship types have a prefix “BKG:” added with them to show that they belong to the BKG. The first line says that we have to look for the entities “x” that belong to the class “Data Source.” The second line says that we have to look for another set of entities “y” that belong to the class Therapeutic or Preventive Procedure. The last line says that “x” (Data Source) should indicate “y” (Therapeutic or Preventive Procedure).

SELECT ?x ?y WHERE {

?x a BKG:Data_Source.

?y a BKG:Therapeutic_or_Preventive_Procedure.

?x BKG:indicates.

?y.}

The above query returns the value “anecdotal report” indicates “famotidine treatment.”

### Extracting Privacy Compliance Requirements From HIPAA COVID-19 and Organizational Privacy Policies

In *Extracting COVID-19 Knowledge From Published Research Paper*, we show how to extract research data related to COVID-19, including data about patients, treatments, and outcomes. It is also necessary to ensure that this data is secured. Any analysis done on shared data should respect associated security and privacy policies. Ensuring security and privacy while processing the data and handling patient records has become a primary challenge. In this section, we describe a framework to extract policy compliance information on data organizations that share and handle COVID-19 data. The policy compliance information is used in association with COVID-19 information extracted from a published research paper in *Making Access Control Decisions to Securely Retrieve COVID-19 Data* to make access control decisions on COVID-19 data. The policy compliance information comes both from individual organization’s privacy policies and HIPAA regulations for COVID-19. We extract knowledge from the organizational privacy policies and HIPAA COVID-19 regulations into a HIPAA ontology. This knowledge graph can be queried to retrieve policy assertions. This, along with other compliance and integrity checks, is used to generate compliance information related to accessing COVID-19 information. The compliance information is eventually used to securely access COVID-19 data.

### Developing HIPAA Ontology

The first step to gathering compliance information is representing policy rules in HIPAA COVID-19 regulation and organizational privacy policy in a knowledge graph. We developed an ontology for the knowledge graph (HIPAA Ontology) to populate extracted policy compliance information from both sources. We describe our method in detail in the following sections.

#### Key Term Extraction

The first stage in developing the HIPAA ontology was to extract key terms from the HIPAA COVID-19 regulation document. In this preprocessing stage, we extracted the rules from the HIPAA document that address COVID-19 regulations. The rules were then analyzed in a bag-of-words model. We removed stop words from the list of words. We also removed certain words like “could,” “shall,” “must,” “will,” “should,” “can.” These modal words were used to extract rules represented in deontic logic from the organizational privacy policies. This is described further in *Extracting Rules From Organizational Privacy Policies*. From the remaining list of words in the HIPAA COVID-19 regulation, we collected the most frequently occurring terms. This list of words is the key terms in the HIPAA repository related to COVID-19. In Table 1 we have listed the top key terms that were extracted from the HIPAA repository related to COVID-19 along with their cumulative frequency. These key terms helped us in generating the HIPAA ontology schema. This is further described in *HIPAA Ontology Schema*. These key terms helped us in checking compliance with organizational privacy policies. This is further discussed in *Frequency of HIPAA Key Terms*.

**TABLE 1:** Key terms from COVID-19 guidance rules from HIPAA.

Keyword	Frequency
HIPAA	56
COVID-19	52
PHI	41
Public	39
Telehealth	36
Provider	25
Communication	21
Notification	20
Individual	19
Privacy	16
Treatment	16
Authorization	16
Remote	15
Disclose	14

#### HIPAA Ontology Schema

**FIGURE 5:**
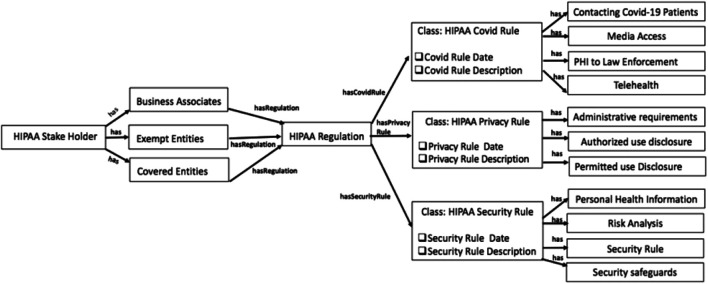
HIPAA regulation knowledge grpah.

Our prior work (Joshi et al., 2016) described a semantically rich knowledge graph with HIPAA rules before COVID-19. In this paper, we expanded the ontology to include COVID-19 updates in the HIPAA regulation. We utilized the key terms extracted in *Key Term Extraction* to define the classes in HIPAA ontology, unlike the manual process we used earlier. These are the key terms in the HIPAA repository related to COVID-19. To build the HIPAA knowledge graph, we used the Prote´ge´ semantic web tool (Protege, 2020). The high-level illustration of entities and relations in the HIPAA Ontology is shown in Figure 5. The primary classes in the HIPAA ontology are as follows:

• The HIPAA Stakeholder class is the main class that signifies the key healthcare providers or organizations who deal with patients’ data and are affected by the health regulations. This class has three main subclasses. These are the Business Associates, Exempt Entities, and Covered Entities. The word “has” means that these are the subclasses associated with a parent class. Each class is disjoint with other classes, which indicates that an individual cannot be an instance of more than one of these three classes.
• The HIPAA Regulation is the top class to describe the regulation and its purpose. Health care providers have requirements that they have to adhere to HIPAA. Business Associates, Exempt Entities, and Covered Entities classes will have a relationship with this class using the object property has Regulation.
• The HIPAA Covid Rule class represents the rules that apply to health care providers that deal or access COVID-19 patients’ data. This class has a relationship with the HIPAA Regulation parent class using the object property has CovidRule. This class has four subclasses Media Access, Contacting Covid-19 patients, PHI to Law Enforcement, and Telehealth indicate various guidance under COVID rules.
• The HIPAA Privacy and Security Rule classes represent privacy and security while accessing health-related data by organizations. We identified these classes and subclasses as part of our previous work (Joshi et al., 2016). Using the object properties has PrivacyRule and has SecurityRule they are associated with the HIPAA Regulation class. Both privacy and security classes have a total of seven classes to describe the associated rules.

#### Extracting Rules From Organizational Privacy Policies

**FIGURE 6:**
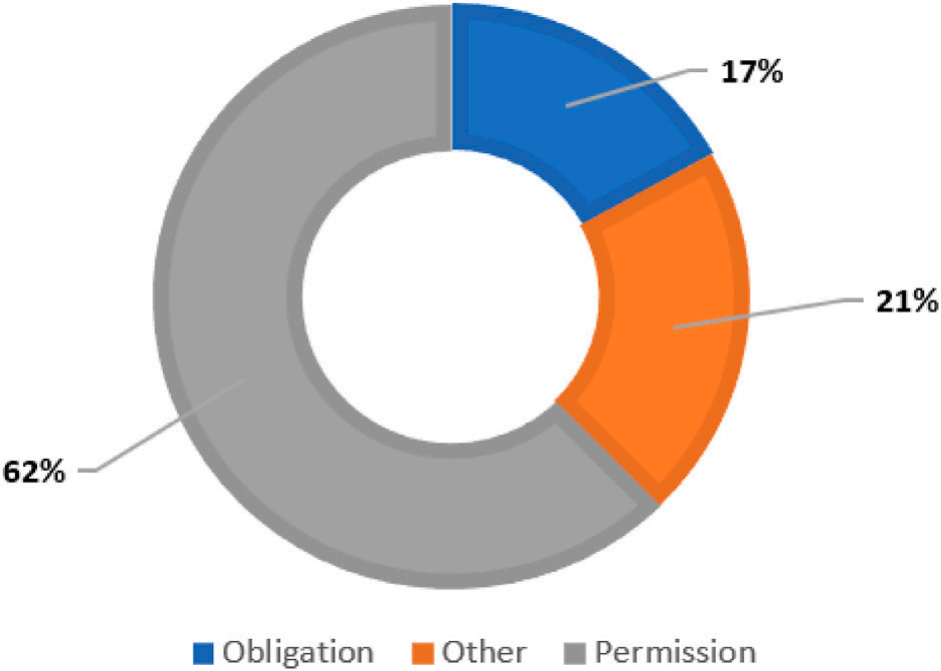
Modal verbs distribution in HIPAA-COVID repository.

In the next step, we populated the HIPAA ontology with rules from organizational privacy policies. In the organizational privacy policy documents, the policy rules are structured as Deontic Logic Statements (DLS). DLS is defined as the statements in a document that express an idea of permission, obligation, dispensation, or prohibition. We utilized DLS to extract policy rules as semi-formal statements from natural language texts. To populate our HIPAA ontology with organizational privacy policy rules, we extracted DLS from the privacy policies that convey a sense of permission or obligation. Figure 6 illustrates the relative frequency of the categories of DLS in the HIPAA COVID guidance.

We utilized modal verbs like “will,” “can,” “could,” “should,” “must” etc for DLS extraction. These modal verbs helped us in identifying DLS in natural language text. They were also used to categorize DLS as permission or an obligation. Sentences with modal verbs like “will,” “can,” “could” are categorized as permissions. Sentences with verbs like “must,” “should” are categorized as obligations. The following are examples of DLS in each category extracted from the HIPAA COVID guidance:

• Permission: “A covered entity may disclose PHI to a first responder who may have been exposed to COVID-19, or may otherwise be at risk of contracting or spreading COVID-19, if the covered entity is authorized by law, such as state law, to notify persons as necessary in the conduct of a public health intervention or investigation” (HHS, 2020).
*•* Obligation: “The covered entity must make reasonable efforts to limit the use or disclosure of PHI to the minimum necessary to accomplish the intended purpose of the use or disclosure” (HHS, 2020).

We extracted the permissions and obligations from organizational privacy policies. The extracted permissions and obligations determined how the HIPAA COVID-19 rules apply to healthcare provider privacy or organizational privacy policies dealing with COVID patient data. The extracted rules were populated into the HIPAA ontology.

### Generating Compliance Information

The HIPAA ontology was developed and populated with policy rules from HIPAA COVID-19 guidance and organization privacy policies. This ontology can be used to retrieve compliance information about organizations dealing with COVID-19 data. In this section, we describe how to retrieve critical compliance information from privacy policies and regulations. In our framework, we used four processes to retrieve the compliance information. We demonstrate these processes by including the results of these evaluations on 10 privacy policies of organizations or health centers dealing with COVID-19 data.

#### Policy Assertion

**FIGURE 7:**
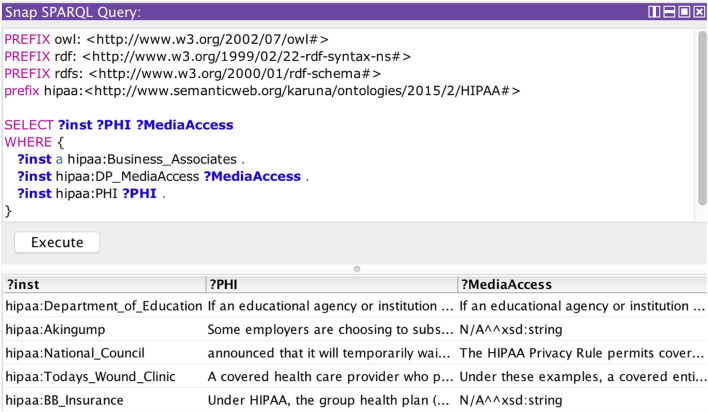
SPARQL query to check for HIPAA COVID rules of privacy policies.

**FIGURE 8:**
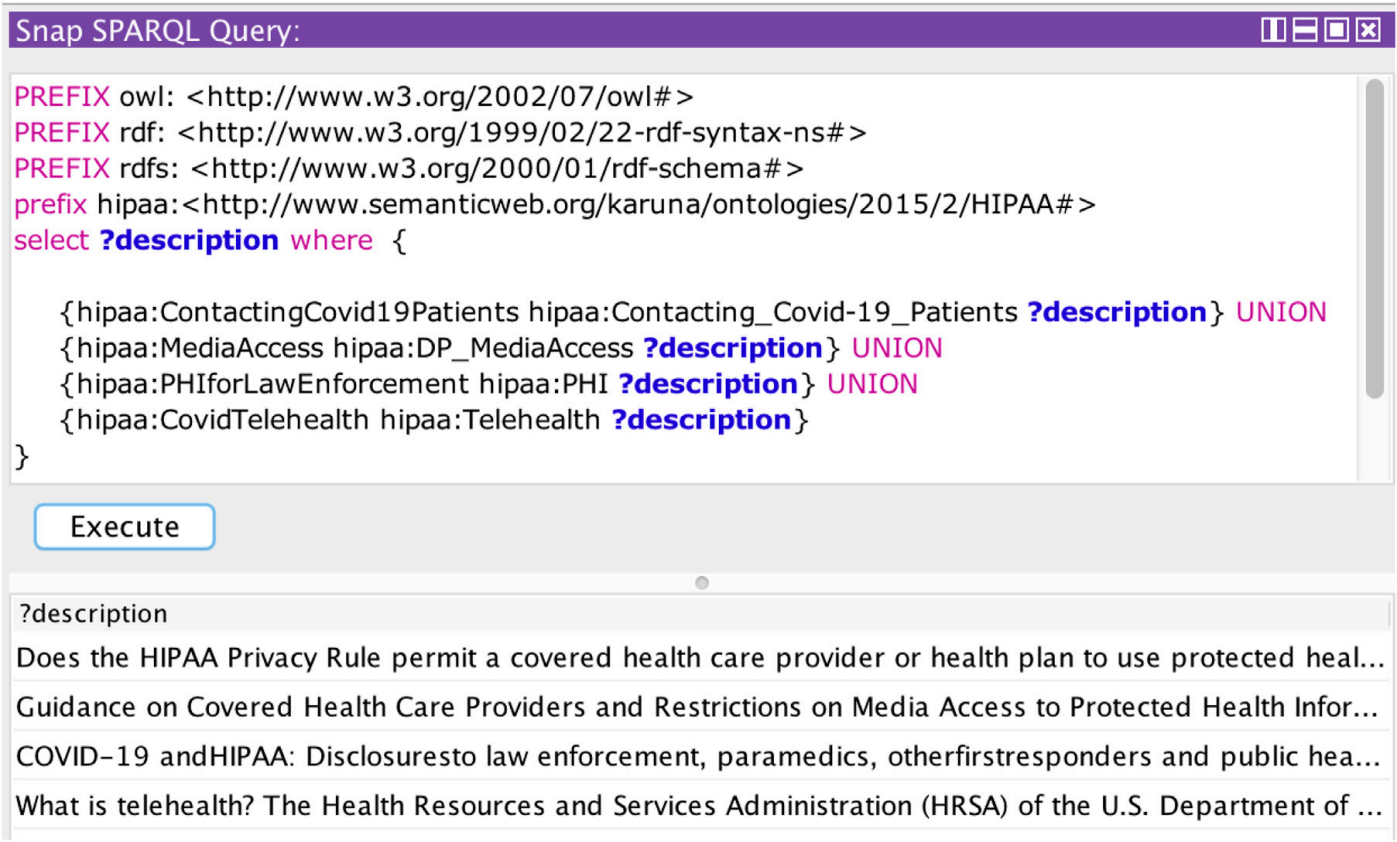
SPARQL query to check for HIPAA COVID rules.

In this section, we demonstrate the reasoning capabilities of the HIPAA ontology and show how it can be used to make policy assertions. We queried the HIPAA ontology using the SPARQL queries (Sirin and Parsia, 2007). Figure 7 demonstrates the query results to check for the HIPAA COVID rules followed by a specific organization. Rules that are missing in the privacy policy are shown as N/A. Figure 8 shows the query results to check all the rules in HIPAA regulation related to COVID-19. Organizations can query the HIPAA ontology to quickly check any rules in HIPAA. Based on this analysis, they can reexamine their policies to address HIPAA guidelines. This automated approach can alert the providers in case of any possible compliance violation. In our framework, we used the policy assertion to check if the organizational policies are compliant with HIPAA regulations. This information forms a part of the compliance information of an organization’s privacy policy.

#### Frequency of HIPAA Key Terms

**FIGURE 9:**
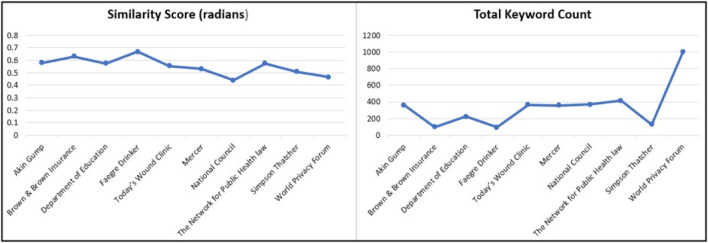
Frequency of semantically similar HIPAA key terms in organizational privacy policies.

As mentioned in *Key Term Extraction*, the key terms are the most important words in the HIPAA COVID-19 regulation. These key terms and words associated with it have to be addressed in an organization’s privacy policy. The frequency of these terms or words related to them is an important indication of a policy’s compliance with the HIPAA guidelines. In our framework, we evaluate the frequency of HIPAA key terms and related terms in a privacy policy. We used the vector representation of key terms to identify semantically similar terms in a document. We illustrate this process by evaluating the frequency of HIPAA key terms in the privacy policies of 10 organizations and health centers that deal with COVID-19 data. Figure 9 shows the frequency of semantically similar HIPAA Key Terms in 10 organizational privacy policies. The higher frequency of HIPAA key terms or semantically similar words in an organization’s privacy policies indicates that the privacy policy is more compliant with the HIPAA regulation. This is one of the compliance information included in our framework.

#### Determining Similarity Score

**FIGURE 10:**
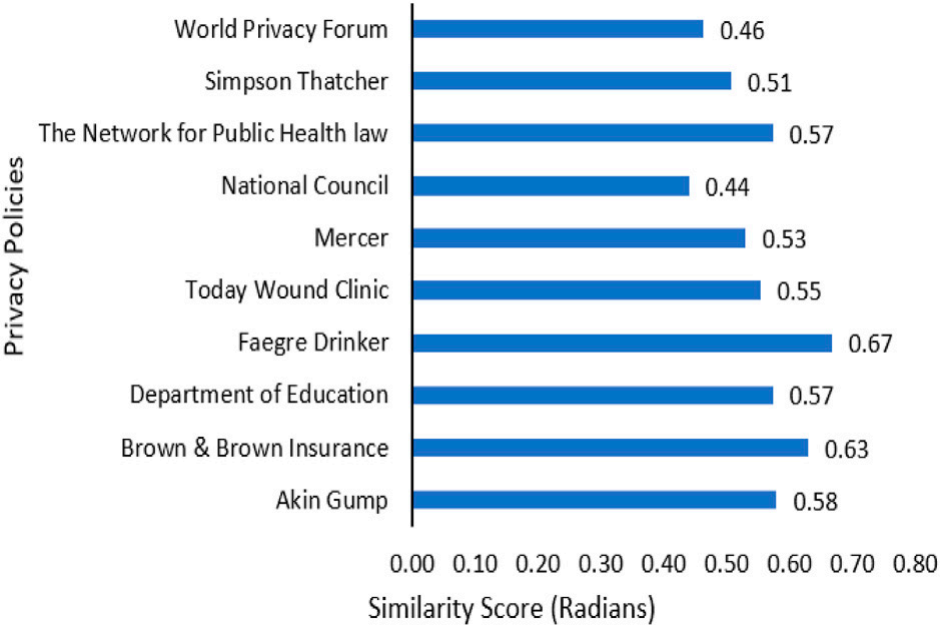
Semantic similarity scores for privacy polices vs. HIPAA COVID rules.

Along with HIPAA key terms, the semantic similarity between the organizational privacy policy and HIPAA regulation is indicative of compliance (Elluri et al., 2020). In our framework, we evaluated the semantic similarity between organizational privacy policies and HIPAA regulation. We included the result of this analysis in the compliance information for the privacy policy. To demonstrate this process, we determined the semantic similarity scores for 10 corpora of ten health provider privacy policies that use COVID-19 data. To measure the semantic similarity, we used the vector representation of the documents in the Doc2Vec model. The similarity score was evaluated in radians. A lower similarity score means that the document is semantically closer to HIPAA and thus more in compliance with the regulation. The results of our analysis for 10 health center privacy policies are illustrated in Figure 10. The similarity scores for these 10 documents are in the range of 0.44–0.67. Organizations like the National Council and World Privacy Forum are more in compliance with the regulation. Five out of ten organizations have an average score of 0.5. An interesting fact is that none of the organizations have higher scores above 0.67, which is essential for health care providers.

#### Assigning Vagueness Score for Privacy Policies

The fourth and final evaluation in our framework to extract compliance information for privacy policies is evaluating the vagueness score for privacy policies. Vagueness or lack of clarity in a text makes it difficult to interpret a text accurately. There are aspects of natural language that allow sentences to be grammatically sound but still unclear in their meaning. If a statement has multiple interpretations and there is no clarification towards the intended meaning, the statement is considered vague. For organizational privacy policies, vagueness contributes to a lack of clarity. This leaves room for misinterpretation. In our framework, we include information about the vagueness of a privacy policy in the compliance information. This helps in deciding the degree to which information extracted from an organizational privacy policy can be trusted.

We analyzed how words and sentence construction choices in English affect the vagueness in statements. We identified three linguistic markers that contribute to vagueness in privacy policy documents. These are:

1. Ambiguous Words: Ambiguous words are the words whose meaning is not clear from the given context. We used the lexical database Wordnet (Miller, 1998) to identify ambiguous words. In Wordnet, words are grouped into sets of cognitive synonyms (synsets), each expressing a distinct concept. If a word is associated with more than one synset, we conclude that it is ambiguous.
2. Vague Words: There are certain words in the English language that are inherently vague. Table 2 provides the taxonomy of vague terms that we used in our model.
3. Reading complexity: The average reading skill of US adults is believed to be at about the 8th-grade level. CalOPPA recommends that privacy policies “be written in clear and concise language, be written at no greater than an 8th-grade reading level.” Overall reading complexity is thus an important measure for lack of clarity in documents. The Dale–Chall readability formula is a readability test that measures the comprehension difficulty that readers face when reading a text.

**TABLE 2:** Categories of vague terms.

Vague terms	
Modal verbs	“may,” “might,” “can”
	“could,” “would,” “likely,” “possible,” “possibly”
Conditional terms	“depending,” “necessary,” “appropriate”
	“inappropriate,” “as needed”
	“as applicable,” “otherwise reasonably,” “sometimes,” “from time to time”
Generalization terms	“generally,” “mostly,” “widely”
	“general,” “commonly,” “usually,” “normally,” “typically,” “largely,” “often,” “primarily,” “among other things”
Generalizing numeric terms	“anyone,” “certain,” “everyone”
	“numerous,” “some,” “most”
	“few,” “much,” “many,” “various,” “including but not limited to”

Using the measures described above, we evaluate the aggregated score of vagueness for a privacy policy. We rescaled the assigned score to the ranges of (1 3). A higher score indicated a privacy policy that is complex and hard to read. A lower score indicated a privacy policy that is relatively easy to read. We used this model to analyze vagueness in privacy policy texts of 10 organizations that collect, store and/or use patients’ data related to COVID-19. The results from our experimental evaluation are provided in Table 3.

**TABLE 3:** Score of vagueness for organization with COVID-19 data.

Organization	Ambiguous words	Vague terms	Reading complexity	Score of vagueness
Akin gump	0.657	0.167	0.98	1.804
Brown and brown insurance	0.602	0.001	0.129	0.732
Department of education	0.667	0.219	0.927	1.813
National council	0.689	0.192	0.471	1.352
Faegre drinker	0.629	0.078	0.889	1.596
Today’ wound clinic	0.669	0.212	1.481	2.362
Mercer	0.638	0.156	0.567	1.361
The network for public health law	0.653	0.096	0.064	0.813
Simpson thatcher	0.657	0.153	0.645	1.455
World privacy forum	0.674	0.225	1.1278	2.0268

### Making Access Control Decisions to Securely Retrieve COVID-19 Data

**FIGURE 11:**
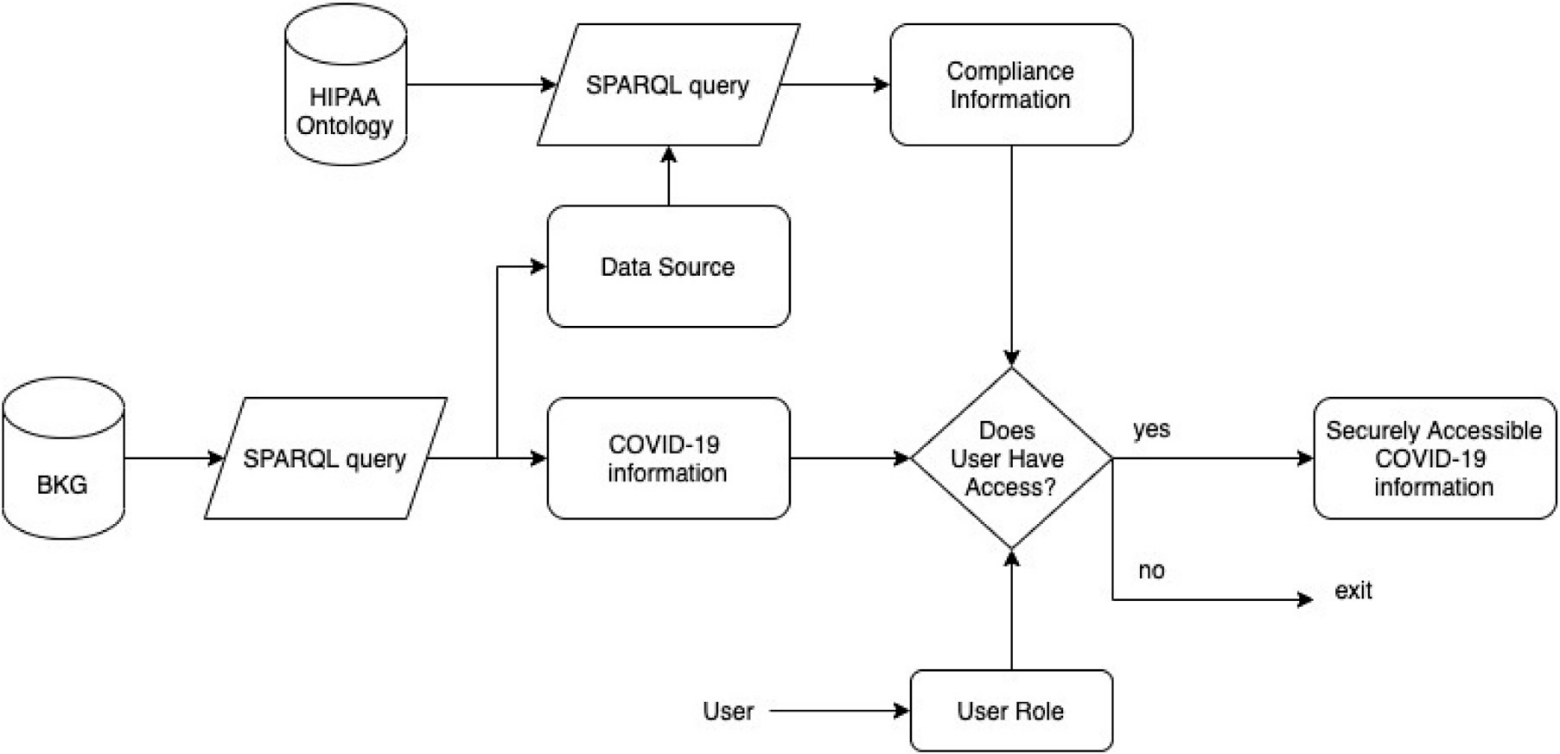
Framework to generate securely accessible COVID-19 data from BKG and HIPAA ontology.

In *Extracting COVID-19 Knowledge From Published Research Paper*, we extracted medical information from COVID-19 research paper. In *Generating Compliance Information*, we extracted compliance information on organizations that collect, share and/or access COVID-19 data. The COVID-19 research paperrely on data sources that adhere to policy regulations like HIPAA. While the research papers are publicly available, extracting data from it and feeding it to a larger KG could potentially leak information that needs to be protected. As recent studies (Vadiya et al.) show, inference attacks can infer data from de-identified sources. This is even more critical for a KG that extracts data from multiple published papers and has reasoning capabilities. The KG can be exploited for an inference attack even though data in individual papers was anonymized. In this section, we show how to use compliance information to restrict access to COVID-19 data and information. Controlling access to information or data is necessary to maintain the integrity and security of data. Figure 11 gives the overview of the framework to securely access COVID-19 data from BKG and HIPAA ontology.

In *Querying BKG to Retrieve COVID-19 Information*, we show how the BKG can be queried using SPARQL to retrieve COVID-19 information from published works. We also give an example of a SPARQL query that can output the data source given a piece of specific information. This data source is one of the organizations that share COVID-19 data. Hence, the corresponding privacy policy for the organization was populated into the HIPAA ontology, *Extracting Rules From Organizational Privacy Policies*, and verified against the HIPAA regulations. The compliance information for the data organization was used to make access control decisions on the COVID-19 information extracted from published works.

We explain with an example the details of the framework. Consider the published work on “Acute Heart Failure in Multisystem Inflammatory Syndrome in Children in the Context of Global SARS-CoV-2 Pandemic” (Belhadjer et al., 2020). In our framework, the data source for the information extracted from this paper was linked to CDC (Covid et al., 2020). We populated the CDC privacy policy^1^ onto the HIPAA ontology. One of the rules that the HIPAA ontology retrieved from the CDC privacy policy was, “We do not use or share your information for commercial purposes and, except as described above, we do not exchange or otherwise disclose this information^2^.” To provide access control, we also queried the users’ role. The access control stage queries, “Is the user a commercial agent?” and “Does the user intend to share this information for commercial purposes?” If the answer to both questions are “No,” the user is allowed access. Else, the user is denied access as they violate the privacy policies for the data. By this framework, we ensure that COVID-19 information is only accessible to agencies who have the right/permission.

## Conclusion and Future Work

Health regulations keep regularly updating, so providers have to update their privacy policies to address the latest rules if they are using patients’ data. Throughout the globe, providers update their privacy policies to demonstrate their commitment to HIPAA compliance and announce a modified edition of their policies by including the context related to the latest regulation rules. Privacy policies are short text and are available in textual format. Therefore, it requires a significant amount of human labor and intervention to ensure compliance with the updated regulation context rules. We anticipate that a semantically rich, machine-processable knowledge graph that captures health provider privacy policies dealing with COVID-19 data will substantially help automate their approach and keep it up to date with any new announcement.

In this paper, we extracted medical information from COVID-19 research papers. We used semantically similar keyword search and text mining to extract compliance information on organizations that collect, share and/or access COVID-19 data. We also showed how to use compliance information to restrict access to COVID-19 data and information. Controlling access to information or data is necessary to maintain the integrity and security of data. In future work, we aim to extract the topics related to short text using deep learning methods and classify them with the topics extracted from the HIPAA regulation.

## Data Availability

The original contributions presented in the study are included in the article/Supplementary Material, further inquiries can be directed to the corresponding author.

## Footnotes

1
https://www.cdc.gov/other/privacy.html

2
https://www.cdc.gov/other/privacy.html#Sharing
